# CircRNA_1156 Attenuates Neodymium Nitrate-Induced Hepatocyte Ferroptosis by Inhibiting the ACSL4/PKCβII Signaling Pathway

**DOI:** 10.3390/antiox14060700

**Published:** 2025-06-09

**Authors:** Ning Wang, Jing Leng, Jing Xu, Kelei Qian, Zhiqing Zheng, Gonghua Tao, Ping Xiao, Xinyu Hong

**Affiliations:** Institute of Chemical Safety Evaluation, State Environmental Protection Key Laboratory of Health Impact Assessment on New Environmental Pollutants, Shanghai Municipal Center for Disease Control and Prevention, Shanghai 201107, China; wangning@scdc.sh.cn (N.W.); lengjing@scdc.sh.cn (J.L.); xujing@scdc.sh.cn (J.X.); qiankelei@scdc.sh.cn (K.Q.); zhengzhiqing@scdc.sh.cn (Z.Z.); taogonghua@scdc.sh.cn (G.T.)

**Keywords:** circRNA_1156, neodymium nitrate, ferroptosis, ACSL4/PKCβII pathway, hepatocyte injury

## Abstract

Ferroptosis, a form of regulated cell death driven by lipid peroxidation, has been implicated in the pathogenesis of liver diseases. This study investigates the role of circRNA_1156 in neodymium nitrate (Nd(NO_3_)_3_)-induced hepatocyte ferroptosis. Our in vitro experiments revealed that exposure to Nd(NO_3_)_3_ (1.2 µM) significantly reduced the viability of AML12 hepatocytes (*p* < 0.01), increased levels of reactive oxygen species (ROS) and malondialdehyde (MDA) (*p* < 0.001), and depleted glutathione (GSH) (*p* < 0.001). However, overexpression of circRNA_1156 effectively reversed these effects and suppressed the expression of ACSL4 and PKCβII (*p* < 0.01). In our in vivo experiments, chronic exposure to Nd(NO_3_)_3_ (7–55 mg/kg for 180 days) induced hepatic iron deposition, mitochondrial damage, and activation of the ACSL4/PKCβII pathway (*p* < 0.01). These adverse effects were significantly ameliorated by circRNA_1156 overexpression (*p* < 0.05). Our findings identify circRNA_1156 as a novel inhibitor of Nd(NO_3_)_3_-induced ferroptosis via downregulation of the ACSL4/PKCβII pathway, providing valuable therapeutic insights for hepatotoxicity caused by rare earth element compounds.

## 1. Introduction

Rare earth elements, due to their distinctive physicochemical attributes, are widely used in industrial, medical, and agricultural fields. However, their potential for biological toxicity has increasingly attracted attention. Neodymium nitrate (Nd(NO_3_)_3_), as a typical rare earth compound, can cause liver damage upon long-term exposure, yet its specific toxicological mechanisms have not been fully elucidated. In recent years, ferroptosis—a form of regulated cell death driven by lipid peroxidation—has been found to play a key role in pathological processes such as heavy metal toxicity, liver fibrosis, and hepatocellular carcinoma [1]. Ferroptosis, a form of regulated cell death driven by lipid peroxidation, is closely associated with oxidative stress, characterized by the depletion of glutathione (GSH), accumulation of lipid reactive oxygen species (ROS), and mitochondrial structural abnormalities. In this study, we measured the levels of ROS (reactive oxygen species), MDA (malondialdehyde), and GSH (glutathione) to evaluate the oxidative stress in AML12 hepatocytes after exposure to Nd(NO_3_)_3_. The core regulatory genes, such as ACSL4 (acyl-CoA synthetase long-chain family member 4) and PKCβII (protein kinase CβII), promote the metabolism of polyunsaturated fatty acids and exacerbate cell death through oxidative stress, becoming a research hotspot in this field [2]. Neodymium, a rare earth element, is predominantly found in regions with rare earth element mining, such as China and the United States. Occupational exposure to neodymium is common in mining and industrial settings. Additionally, neodymium is widely used in electronics and medical devices, increasing the potential for environmental and occupational exposure. Given the increasing socioeconomic importance of rare earth elements, understanding their potential health impacts is crucial. This study aims to elucidate the role of circRNA_1156 in neodymium nitrate (Nd(NO_3_)_3_)-induced hepatocyte ferroptosis. Its core regulatory genes, such as ACSL4 (acyl-CoA synthetase long-chain family member 4) and PKCβII (protein kinase CβII), promote the metabolism of polyunsaturated fatty acids and exacerbate cell death through oxidative stress, becoming a research hotspot in this field [3].

In addition to neodymium, other rare earth elements (REEs) have also been implicated in various health issues. For instance, a significant increase in the incidence of respiratory and liver diseases in communities exposed to rare earth elements was reported, particularly in mining regions. This study highlights the potential health risks associated with occupational and environmental exposure to REEs. Epidemiological studies have further underscored the adverse health effects of REEs, linking chronic exposure to increased rates of chronic obstructive pulmonary disease (COPD) and liver fibrosis. These findings emphasize the need for comprehensive research to understand the mechanisms underlying REE-induced health impacts.

Given the increasing socioeconomic importance of rare earth elements and the emerging evidence of their potential health impacts, understanding the specific mechanisms by which these elements induce toxicity is crucial. Our findings identify circRNA_1156 as a novel inhibitor of Nd(NO_3_)_3_-triggered ferroptosis via ACSL4/PKCβII downregulation, offering therapeutic insights for rare earth element compound-induced hepatotoxicity.

circRNA_1156, a specific type of circRNA, was found to be significantly downregulated in hepatocytes exposed to Nd(NO_3_)_3_ in recent high-throughput sequencing studies [4,5,6]. This finding suggests that circRNA_1156 may play a crucial role in the cellular stress response induced by Nd(NO_3_)_3_. Based on its expression changes under specific conditions, we propose the following hypothesis: circRNA_1156 may influence ferroptosis by regulating the ACSL4/PKCβII pathway. This hypothesis is based on the diverse functional mechanisms of circRNA within cells, including acting as a miRNA sponge to regulate gene expression and interacting with other RNA-binding proteins to affect protein function and localization.

To verify this hypothesis, we plan to conduct a series of in vitro and in vivo experiments in our subsequent research to thoroughly explore the specific mechanisms by which circRNA_1156 affects ferroptosis. This will involve a detailed analysis of the interactions between circRNA_1156 and molecules related to the ACSL4/PKCβII pathway, as well as an evaluation of how changes in circRNA_1156 expression impact the ferroptosis process in cells [7,8,9]. Through these studies, we aim to clarify the potential regulatory mechanisms of circRNA_1156 in ferroptosis, providing new strategies and targets for the treatment of related diseases [10,11,12].

Based on this, the present study aims to investigate the regulatory role of circRNA_1156 in Nd(NO_3_)_3_-induced ferroptosis using AML12 hepatocytes and C57BL/6J mice as models. By combining cell viability assays, oxidative stress analysis, and molecular biological techniques, we systematically elucidate the regulatory mechanisms of circRNA_1156 on the ACSL4/PKCβII pathway, with the goal of revealing new targets for rare earth element-induced hepatotoxicity and providing a theoretical basis for liver protection strategies [13,14,15].

A detailed overview of the experimental design is provided in Figure S1 (Experimental Design Flowchart).

## 2. Materials and Methods

### 2.1. Cell Culture and Treatments

#### 2.1.1. Cell Culture and Treatment

Normal mouse hepatocytes (AML12 cells, strain GNM42) obtained from the Chinese Academy of Sciences Cell Bank (Shanghai, China) were cultured in DMEM/F12 medium (Gibco, 12634010, Thermo Fisher Scientific, Waltham, MA, USA) supplemented with 10% fetal bovine serum (FBS, 10099141C, Gibco, Thermo Fisher Scientific, Scoresby, Australia) in a humidified incubator at 37 °C with 5% CO_2_. The AML12 cells were randomly divided into four groups: the control group, the Nd(NO_3_)_3_ treatment group (1.2 µM Nd(NO_3_)_3_ (Sigma-Aldrich, 269885, St. Louis, MO, USA)), the circRNA_1156 overexpression plasmid transfection group, and the group treated with both the circRNA_1156 overexpression plasmid and Nd(NO_3_)_3_. The circRNA_1156 overexpression plasmid was purchased from Hangzhou Guannan Biotechnology Co., Ltd. (Hangzhou, China). The cells were transfected with the circRNA_1156 overexpression plasmid using Lipofectamine 3000 reagent (Thermo Fisher, L3000015, Waltham, MA, USA) according to the manufacturer’s instructions. After 24 h of transfection, the cells were treated with Nd(NO_3_)_3_ for an additional 24 h.

Plasmid Construction: The full-length sequence of circRNA_1156 (5′-GTAAGGTGTGGCTTCTGGCTCAGTTGGAAAGGTGCAAGGC-3′) was cloned into a lentiviral or plasmid vector (such as pCDH-75 CMV-MCS-EF1-Puro). The vector was ensured to contain a selection marker (such as puromycin resistance) and a circRNA-specific promoter. Lipofectamine 3000 (Thermo Fisher, L3000015, Waltham, MA, USA) was used to transfect AML12 cells, and the overexpression efficiency was verified by qPCR.

#### 2.1.2. Cell Viability Assay

AML12 hepatocytes were treated with various concentrations of Nd(NO_3_)_3_ (0.4 µM, 0.8 µM, 1.2 µM, and 1.6 µM) for different time periods (24 h and 48 h). The primary concentration used was 1.2 µM for 24 h, as it provided the most significant results. Additional concentrations and time points were tested to establish a dose–response and time-course relationship. The viability of the cells was assessed using the Cell Counting Kit-8 (CCK-8) assay (Beyotime, C0038, Shanghai, China) according to the manufacturer’s instructions. The absorbance was measured at 450 nm using a microplate reader (Tecan, SPARK30086376, Männedorf, Switzerland).

#### 2.1.3. Detection of Intracellular ROS, Mitochondrial ROS, and Mitochondrial Membrane Potential

Cells were cultured in 6-well plates and treated as described above. After reaching the designated time points, the following assays were performed to detect intracellular ROS, mitochondrial ROS, and mitochondrial membrane potential.

Detection of Intracellular ROS: 2′,7′-dichlorofluorescin diacetate (DCFH-DA, 10 µM) (MedChemExpress, HY-101100, Shanghai, China) was used as the fluorescent probe for intracellular ROS. Cells were washed once with PBS, then incubated with DCFH-DA (10 µM) in serum-free medium for 20 min at 37 °C in the dark. Cells were washed twice with PBS to remove unincorporated DCFH-DA and reduce background fluorescence. Fluorescence was detected using a fluorescence microscope (Leica DM3000, Leica Microsystems, Wetzlar, Germany) with an excitation wavelength of 488 nm and an emission wavelength of 525 nm. The intensity of the fluorescence indicated the level of intracellular ROS.

Detection of Mitochondrial ROS: After measuring intracellular ROS, the cells were harvested and transferred to a 1.5 mL centrifuge tube after washing once with PBS. An appropriate amount of hypotonic buffer was added, and cells were incubated on ice for approximately 1 h, mixing occasionally. A Dounce homogenizer was used to manually homogenize the cells. The cell viability was checked with Trypan Blue during the process. Homogenization was stopped when approximately 50% of the cells were broken. Then, cells were centrifuged at 3000 rpm (approximately 1000× *g*) for 10 min at 4 °C. The supernatant was transferred to a new tube. Centrifugation was continued at 12,000 rpm for 15 min. The pellet obtained was the mitochondrial fraction. Mitochondrial ROS was measured using the same method as for intracellular ROS detection.

Detection of Mitochondrial Membrane Potential: A mitochondrial membrane potential assay kit with Rhodamine 123 (Beyotime, C1052, Shanghai, China) was used. Cells were washed once with PBS, then incubated with Rhodamine 123 (1 µM) in serum-free medium for 30 min at 37 °C in the dark. Cells were washed twice with PBS to remove unincorporated Rhodamine 123 and reduce background fluorescence. Fluorescence was detected using a fluorescence microscope (Leica DM3000, Leica Microsystems, Wetzlar, Germany) with an excitation wavelength of 488 nm and an emission wavelength of 525 nm. The intensity of the fluorescence indicates the mitochondrial membrane potential. A decrease in fluorescence intensity indicated a loss of mitochondrial membrane potential, which is often associated with cell apoptosis or oxidative stress.

#### 2.1.4. Cellular Ferroptosis Marker Assays

Cell lysates or supernatants from tissue homogenates were used for the assays. Protein concentrations were determined using a BCA Protein Assay Kit (Beyotime, P0012S, Shanghai, China) to generate a standard curve. The protein concentration in each sample was calculated based on the standard curve and normalized accordingly.

The levels of superoxide dismutase (SOD) were measured using an SOD assay kit (Beyotime, S0109, Shanghai, China). The assay was performed according to the manufacturer’s protocol, which involved the reduction of nitroblue tetrazolium (NBT) by SOD in the presence of superoxide anions to form a blue-colored formazan product. The absorbance was measured at 560 nm.

The levels of malondialdehyde (MDA) were measured using an MDA assay kit (Beyotime, S0131S, Shanghai, China) according to the manufacturer’s instructions. Briefly, tissue or cell lysates were prepared, and protein concentrations were determined using a BCA Protein Assay Kit. The MDA levels were then quantified by measuring the absorbance at 532 nm.

The levels of glutathione (GSH) were measured using a GSH assay kit (Beyotime, S0052, Shanghai, China). The assay was performed according to the manufacturer’s protocol, which involved the reaction of GSH with DTNB (5,5′-dithiobis-2-nitrobenzoic acid) to produce a yellow-colored product. The absorbance was measured at 412 nm.

#### 2.1.5. Real-Time Quantitative PCR

The primer sequences were derived from the high-throughput sequencing data obtained in the preliminary study. The specificity of the primers was validated using NCBI Primer-BLAST (Version 2.2.29, NCBI, Bethesda, MD, USA) to ensure that the amplification target was the circular junction site of circRNA_1156 (forward primer: GTAAGGTGTGGCTTCTGGCT; reverse primer: CAGTTGGAAAGGTGCAAGGC). Design Parameters: The GC content was maintained between 45% and 55%, the melting temperature (Tm) was approximately 60 °C, and the design avoided primer dimers and nonspecific binding.

Total RNA was isolated from tissues or cells with TRIzol reagent (Tiangen, DP424, Beijing, China), followed by reverse transcription into cDNA using the FastKing One-Step RT-PCR Kit (Tiangen, KR123, Beijing, China). Quantitative real-time PCR (qRT-PCR) was performed on an Mx3000P Real-Time PCR System (Jiatai, Shanghai, China) using SuperReal PreMix Plus (SYBR Green, Tiangen, FP215, Beijing, China). Specific primers for the target genes are listed in Table 1. The relative expression of mRNAs was normalized to β-actin.

#### 2.1.6. Western Blot Analysis

Proteins were isolated from tissues or cells using a lysis buffer (Thermo Fisher, 89900, Waltham, MA, USA). The concentration of the extracted proteins was determined using a BCA Protein Assay Kit (Beyotime, P0012S, Shanghai, China). The proteins were then denatured and separated by SDS-PAGE, followed by transfer to PVDF membranes (Millipore, IPVH00010, Billerica, MA, USA) via electroblotting. The membranes were blocked with 5% skim milk at room temperature for 1 h and subsequently incubated with primary antibodies targeting ACSL4 (Beyotime, AG1908, Shanghai, China; 1:1000 dilution), PKCβII (Beyotime, AF1768, Shanghai, China; 1:1000 dilution), and LPCAT1 (Beyotime, AG5215, Shanghai, China; 1:1000 dilution) at 4 °C overnight (Table 2). After washing, the membranes were incubated with secondary antibodies (diluted 1:1000, Beyotime, A0208, Shanghai, China) for 1 h at room temperature. The intensity of the protein bands was analyzed using ImageJ software (Version: 1.53t, National Institutes of Health, Bethesda, MD, USA).

### 2.2. In Vivo Experimental Procedures

#### 2.2.1. Animal Housing and Administration

Six-week-old male C57BL/6J mice (weighing 21–23 g) of specific pathogen-free (SPF) grade were obtained from Zhejiang Vitonliva Biotechnology Co., Ltd. (Hangzhou, China). After a 1-week acclimatization period, the mice were randomly assigned to six groups (water control group, Nd(NO_3_)_3_-7, 14, 27, 39, and 55 mg/kg), with six animals per group (*n* = 6). The corresponding concentrations of neodymium nitrate hexahydrate solution used for oral gavage were 0.9, 1.8, 3.6, 5.2, and 7.3 mg/mL at a dosage of 10 mL/kg body weight once daily. On day 180, the mice from each group were sacrificed, and blood and liver tissues were collected for subsequent evaluation. The mice were euthanized by cervical dislocation under anesthesia (isoflurane, Solebo, 25 g, Beijing, China) on day 180 of the experiment. This method was chosen to minimize animal suffering and ensure a quick and humane death. This study was approved by the Animal Ethics Committee of the Shanghai Center for Disease Control and Prevention and conducted in accordance with the National Institutes of Health Guide for the Care and Use of Laboratory Animals.

#### 2.2.2. Body Weight Measurement

Body weights were measured once a week during the experimental period.

#### 2.2.3. Liver Weight and Organ Index

At the end of the experiment, all animals (*n* = 6 per group) were subjected to gross dissection. The absolute weight of the liver was recorded, and the relative weight (liver-to-body weight ratio) was calculated.

#### 2.2.4. Hematoxylin and Eosin (H&E) Staining

Liver tissues from mice with chronic liver injury induced by Nd(NO_3_)_3_ were processed for histological analysis. For histological analysis, three liver sections per mouse were prepared and stained with hematoxylin and eosin (H&E). The tissues were fixed in 4% paraformaldehyde (Senbeiga, BL-G002, Nanjing, China) overnight, followed by dehydration in a graded ethanol series and embedding in paraffin. Paraffin-embedded tissue blocks were sectioned at a thickness of 5 µm. The sections were then stained with hematoxylin (Solarbio, H8070, Beijing, China) and eosin (Sangon, A600190, Shanghai, China) to visualize cellular structures. The stained sections were examined and imaged using a light microscope (Leica DM3000, Wetzlar, Germany).

#### 2.2.5. Prussian Blue Staining

Prussian blue staining (Servicebio, G1029, Wuhan, China) was used to observe iron deposition in liver tissues. For Prussian blue staining analysis, three liver sections per mouse were prepared and stained with Prussian blue. Prussian blue staining solution was added to the prepared liver tissue sections for 1 h. After rinsing with distilled water, Prussian blue staining solution was added and stained for 3 min. Finally, the sections were washed with water until the effluent was colorless, dehydrated with anhydrous ethanol, and sealed with neutral gum. The staining effect was observed and photographed under a microscope (Leica DM3000, Leica Microsystems, Wetzlar, Germany).

#### 2.2.6. Fluorescence In Situ Hybridization (FISH) Experiment

To design a probe for circRNA_1156, the sequence should specifically target the circular junction site, such as the back-splicing site, to avoid overlap with linear RNA. The probe should be 20–30 nucleotides in length, with a GC content of around 50%, and its specificity should be confirmed using BLAST (Version 2.2.29, NCBI, Bethesda, MD, USA). For the experiment, an RNASweAMI™ CY3-labeled probe (e.g., 5′-CY3-XXX-3′) can be used combined with a sequence specific to circRNA_1156. The consistency of the probe signal should be validated against qPCR results.

Liver tissue samples from mice with chronic liver injury induced by Nd(NO_3_)_3_ were prepared as frozen sections. For fluorescence in situ hybridization (FISH) staining, five frozen sections per mouse were used. The procedure followed the instructions of the RNASweAMI™ In Situ Hybridization CY3 Detection Kit (Servicebio, GF002-50T, Wuhan, China). The frozen sections were thawed at room temperature for 10 min, then fixed with the kit’s fixative for 10 min at room temperature. After drying, the sections were washed three times with PBS (5 min each). An immunohistochemistry pen was used to outline the tissue, and Proteinase K working solution was added to cover the tissue. The sections were incubated at 37 °C for 5 min, followed by three washes with PBS (5 min each). The sections were then placed in a humidified chamber, and preheated hybridization buffer (40 °C, approximately 60 µL) was added to cover the samples. The sections were incubated at 40 °C for 30 min. The hybridization buffer was replaced with preheated target probe mixture 1 hybridization buffer (40 °C, approximately 60 µL), and the sections were incubated at 40 °C for 3 h (or overnight at 40 °C). The sections were then washed sequentially with preheated 2 × SSC, 1 × SSC, 0.5 × SSC, and 0.1 × SSC (5 min each at 40 °C). The process was repeated with target probe mixture 2 hybridization buffer (40 °C, approximately 60 µL) for 45 min, followed by the same washing steps. Finally, preheated fluorescent signal probe hybridization buffer (37 °C, approximately 60 µL) was added, and the sections were incubated at 37 °C for 3 h. The sections were washed again with preheated 2 × SSC, 1 × SSC, 0.5 × SSC, and 0.1 × SSC (5 min each at 37 °C) and rinsed once with 1×PBS. DAPI working solution (200–500 µL) was added to stain the nuclei in the dark at room temperature for 8 min. Without washing, the sections were dried slightly and mounted with an antifade medium. The sections were observed under a fluorescence microscope (Leica DM3000, Leica Microsystems, Wetzlar, Germany) at 40× magnification with appropriate fluorescence filters. For fluorescence intensity comparison, the microscope acquisition parameters were kept consistent, and fluorescence intensity was analyzed using ImageJ software.

#### 2.2.7. Immunofluorescence Staining of Frozen Sections

Liver tissue samples from mice with chronic liver injury induced by Nd(NO_3_)_3_ were prepared as frozen sections. For immunofluorescence staining, five frozen sections per mouse were used. The sections were fixed in 4% paraformaldehyde (Senbeiga, BL-G002, Nanjing, China) at room temperature for 30–60 min, then washed three times with PBS for 5 min each. The sections were permeabilized with 0.3% Triton X-100 (diluted in PBS) at room temperature for 15 min, followed by three 5 min washes with PBS. The sections were then blocked with an immunostaining blocking solution (Beyotime, P0102, Shanghai, China) at 37 °C for 2 h (no washing required afterward).

Primary antibodies were prepared using the immunostaining primary antibody dilution buffer (Beyotime, P0103, Shanghai, China). The antibodies used were GPX4 Rabbit Monoclonal Antibody (Beyotime, RM5359, 1:200, Shanghai, China), ACSL4 Rabbit Monoclonal Antibody (Beyotime, AG1908, 1:200, Shanghai, China), LPCAT1 Rabbit Monoclonal Antibody (Beyotime, AG5215, 1:200, Shanghai, China), and PKCβII Rabbit Monoclonal Antibody (Beyotime, AF1768, 1:200, Shanghai, China). The sections were circled with a blocking pen to confine the tissue area, and a small amount of antibody was applied to the sections, which were then incubated overnight at 4 °C in a humidified chamber. The sections were washed three times with PBS for 5 min each.

The sections were then incubated with an anti-rabbit FITC immunofluorescence staining kit (Beyotime, P0186, Shanghai, China) in the dark at 37 °C for 2 h, followed by three 5 min washes with PBS. The nuclei were stained with 2 µg/mL DAPI (Servicebio, G1012, Wuhan, China) and washed three times with PBS for 5 min each. The sections were then mounted with an antifade mounting medium.

The sections were observed under a fluorescence microscope (Leica DM3000, Leica Microsystems, Wetzlar, Germany). Fluorescence signals were detected under the appropriate magnification (40×) objective lens through the corresponding fluorescence filters. For images that need to compare fluorescence intensity, it is essential to ensure that the acquisition parameters of the fluorescence microscope are kept consistent. Fluorescence intensity statistics can be performed using ImageJ (Version: 1.53t, National Institutes of Health, Bethesda, MD, USA).

#### 2.2.8. Tissue Ferroptosis Marker Assays

For the assays, cell suspensions or supernatants from tissue homogenates were utilized. The BCA Protein Assay Kit (Beyotime, P0012S, Shanghai, China) was employed to create a protein standard curve, which was subsequently used to determine and normalize the protein concentration in each sample. Additionally, the levels of superoxide dismutase (SOD), malondialdehyde (MDA), and glutathione (GSH) were assessed using specific assay kits for SOD, MDA, and GSH (all sourced from Beyotime, Shanghai, China).

#### 2.2.9. Transmission Electron Microscopy

Tissue samples were collected and fixed in the electron microscopy fixative (Servicebio, G1102, Hubei, China) in the dark at room temperature for 2 h. The samples were then dehydrated with acetone and polymerized in an oven at 60 °C for 48 h. Ultrathin sections (60–80 nm) were cut and stained with 2% saturated uranyl acetate solution and 2.6% lead citrate solution. Mitochondrial morphological changes were observed under a transmission electron microscope (HT7700 Exalens, Hitachi, Tokyo, Japan).

### 2.3. Statistical Analysis

Statistical analysis was performed using GraphPad Prism 8 (Version: 8.4.2, GraphPad Software, San Diego, CA, USA), with results presented as the mean ± standard error (SE). To evaluate differences among multiple groups, one-way analysis of variance (ANOVA) was conducted, followed by Tukey’s HSD test for pairwise comparisons against the control group. A significance level of *p* < 0.05 was applied.

## 3. Results

### 3.1. In Vitro Experimental Results

**Figure 1:**
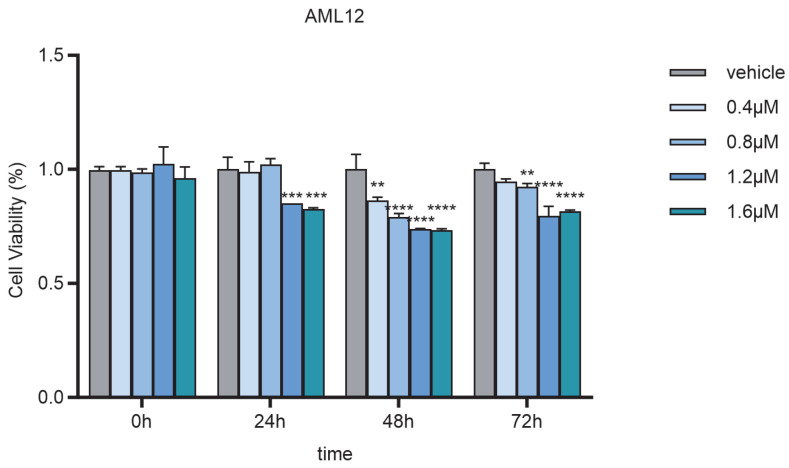
Effect of Nd(NO_3_)_3_ on the viability of AML12 cells. Cell viability was assessed using the CCK-8 assay after 24 h of exposure to Nd(NO_3_)_3_ (1.2 µM). Data are presented as the mean ± SE (*n* = 3 independent experiments). ** *p* < 0.01, *** *p* < 0.001, **** *p* < 0.0001 compared to the control group.

**Figure 2:**
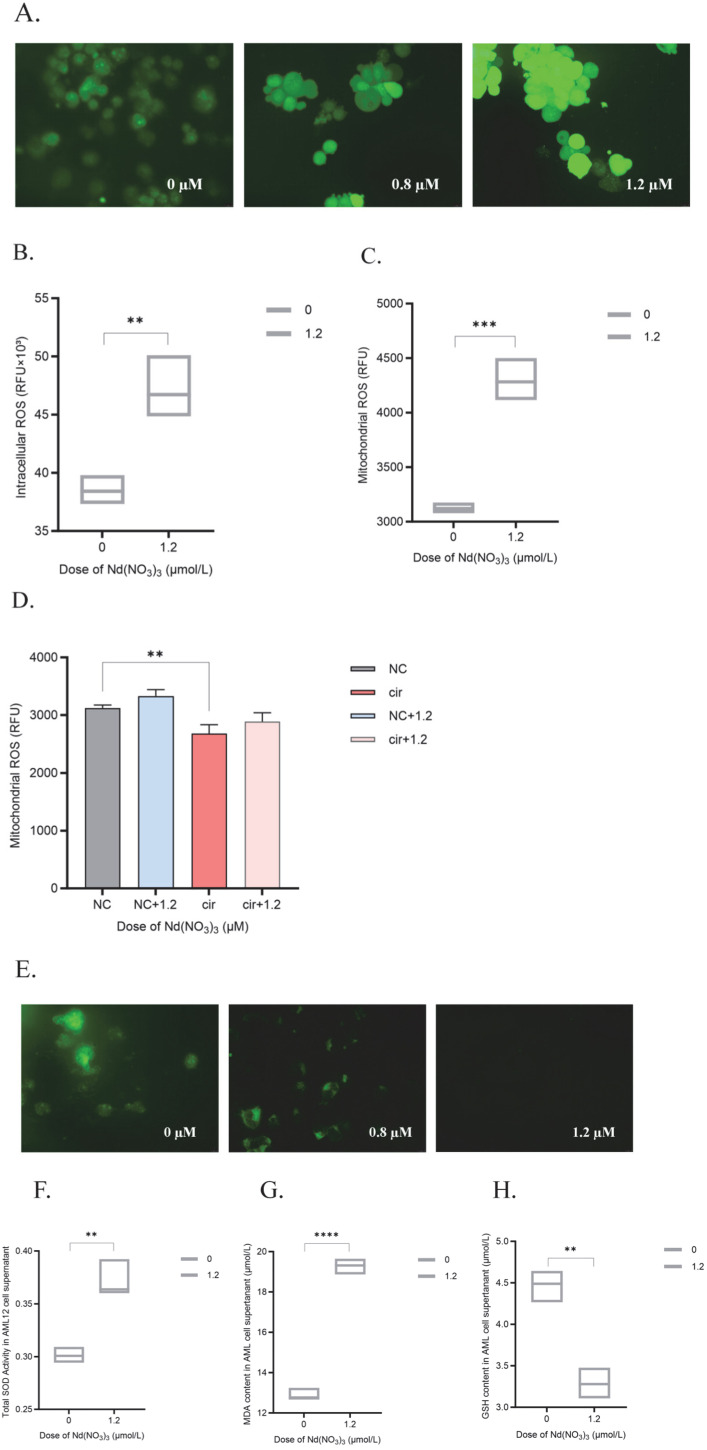
Detection of intracellular ROS, mitochondrial ROS, and ferroptosis-related markers in AML12 cells after 24-h exposure to 1.2 µM Nd(NO_3_)_3_. (**A**) Detection of intracellular ROS using the DCFH-DA probe. (**B**) Quantitative analysis of intracellular ROS. (**C**) Quantitative analysis of mitochondrial ROS in control and circRNA_1156 overexpression cells. (**D**) Quantitative analysis of mitochondrial ROS. (**E**) Measurement of mitochondrial membrane potential (Rhodamine 123). (**F**) Determination of superoxide dismutase (SOD) levels. (**G**) Determination of malondialdehyde (MDA) levels. (**H**) Determination of glutathione (GSH) levels. Data are presented as the mean ± SE (*n* = 3 independent experiments). ** *p* < 0.01, *** *p* < 0.001, **** *p* < 0.0001 compared to the control group.

Effect of Nd(NO_3_)_3_ on AML12 Cell Viability: The primary experimental condition used in this study was 1.2 µM Nd(NO_3_)_3_ exposure for 24 h, as this concentration effectively induced significant ferroptosis in AML12 hepatocytes without causing complete cell death. Under this condition, the viability of AML12 hepatocytes was significantly inhibited, as determined by one-way ANOVA (*F* (3, 8) = 0.01, *p* = 0.986; *F* (3, 8) = −0.02, *p* = 0.868; *F* (3, 8) = 0.15, *p* = 0.0008; *F* (3, 8) = 0.17, *p* = 0.0003, as shown in Figure 1). To establish a comprehensive dose–response and time-course relationship, additional experiments were conducted with Nd(NO_3_)_3_ concentrations ranging from 0.4 µM to 1.6 µM and exposure times up to 48 h. Meanwhile, the levels of intracellular ROS and mitochondrial ROS were significantly elevated (*t* (4) = 5.14, *p* = 0.007; *t* (4) = 10.21, *p* = 0.0005, Figure 2A–C), accompanied by a decrease in mitochondrial membrane potential (Figure 2E).

Mitochondrial ROS Levels in CircRNA_1156 Overexpression Cells: To further investigate the role of circRNA_1156 in modulating oxidative stress, we measured mitochondrial ROS levels in cells overexpressing circRNA_1156. As shown in Figure 2D, overexpression of circRNA_1156 significantly reduced mitochondrial ROS levels compared to control cells (*F* (3, 8) = −208.3, *p* = 0.17; *F* (3, 8) = 445.7, *p* = 0.0059; *F* (3, 8) = 234.7, *p* = 0.12). This reduction in mitochondrial ROS indicates that circRNA_1156 overexpression can mitigate oxidative stress, potentially by downregulating the ACSL4/PKCβII pathway.

**Figure 3:**
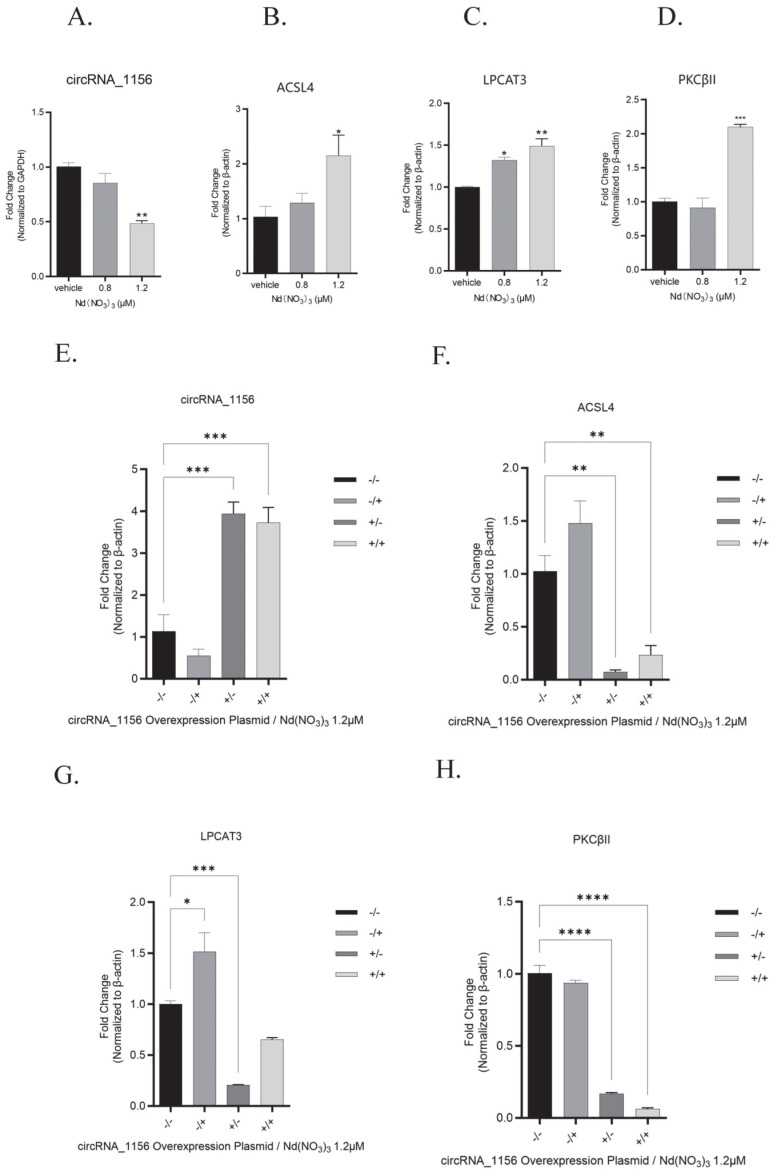
The effect of Nd(NO_3_)_3_ on ferroptosis-related gene expression in AML12 cells. (**A**) Gene expression of circRNA_1156. (**B**) Gene expression of ACSL4. (**C**) Gene expression of LPCAT3. (**D**) Gene expression of PKCβII. (**E**) Gene expression of circRNA_1156 after transfection with circRNA_1156 overexpression plasmid. (**F**) Gene expression of ACSL4 after transfection with circRNA_1156 overexpression plasmid. (**G**) Gene expression of LPCAT3 after transfection with circRNA_1156 overexpression plasmid. (**H**) Gene expression of PKCβII after transfection with circRNA_1156 overexpression plasmid. Legend: “−/−”: No circRNA_1156 overexpression plasmid transfection and no 24-h exposure to 1.2 µM Nd(NO_3_)_3_ (double negative control); “−/+”: No circRNA_1156 overexpression plasmid transfection, but with 24-h exposure to 1.2 µM Nd(NO_3_)_3_ (single treatment control); “+/−”: circRNA_1156 overexpression plasmid transfection without 24-h exposure to 1.2 µM Nd(NO_3_)_3_ (single genetic manipulation group); “+/+”: circRNA_1156 overexpression plasmid transfection plus 24-h exposure to 1.2 µM Nd(NO_3_)_3_ (full treatment group). Data are presented as the mean ± SE (*n* = 3 independent experiments). * *p* < 0.05, ** *p* < 0.01, *** *p* < 0.001, **** *p* < 0.0001 compared to the control group.

**Figure 4:**
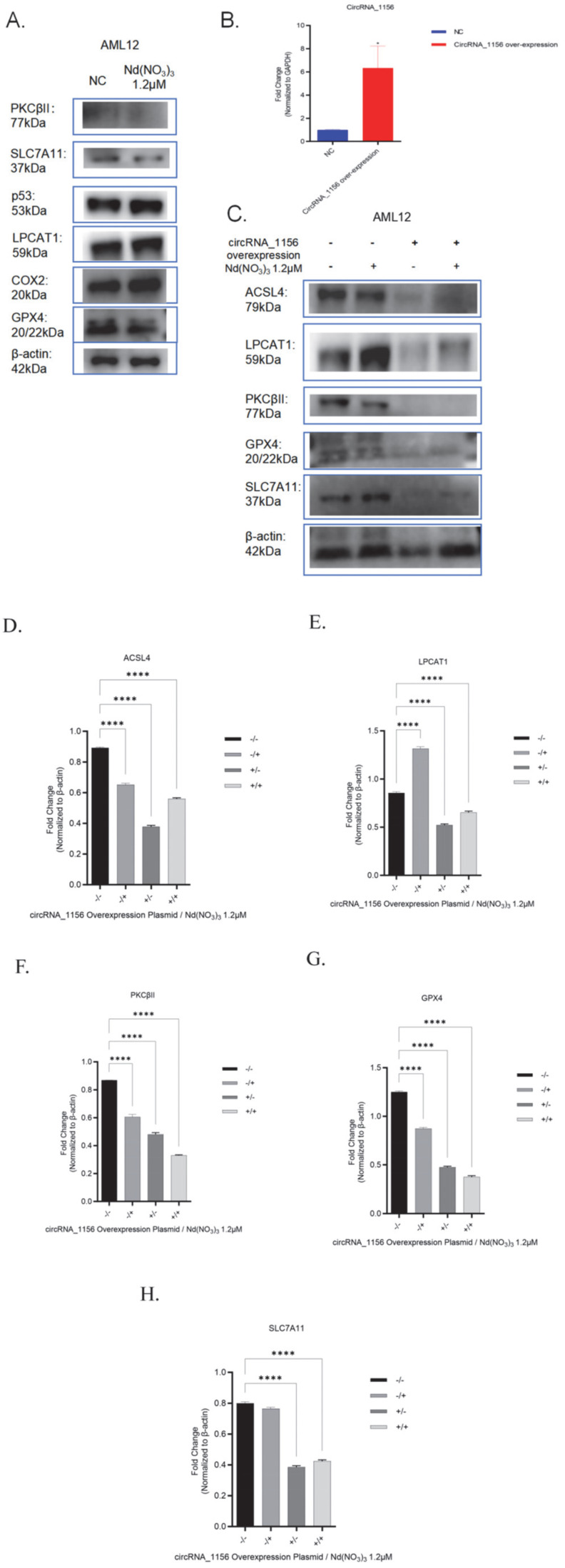
Effects of Nd(NO_3_)_3_ on the expression of ferroptosis-related proteins in AML12 cells. (**A**) Expression of ferroptosis-related proteins in AML12 cells treated with Nd(NO_3_)_3_. (**B**) Gene expression of circRNA_1156 after transfection with circRNA_1156 overexpression plasmid. (**C**) Expression of ferroptosis-related proteins in AML12 cells after transfection with circRNA_1156 overexpression plasmid. (**D**) Grayscale analysis of ACSL4 protein. (**E**) Grayscale analysis of LPCAT1 protein. (**F**) Grayscale analysis of PKCβII protein. (**G**) Grayscale analysis of GPX4 protein. (**H**) Grayscale analysis of SLC7A11 protein. Legend: “−/−”: No circRNA_1156 overexpression plasmid transfection and no 24-h exposure to 1.2 µM Nd(NO_3_)_3_ (double negative control); “−/+”: No circRNA_1156 overexpression plasmid transfection, but with 24-h exposure to 1.2 µM Nd(NO_3_)_3_ (single treatment control); “+/−”: circRNA_1156 overexpression plasmid transfection without 24-h exposure to 1.2 µM Nd(NO_3_)_3_ (single genetic manipulation group); “+/+”: circRNA_1156 overexpression plasmid transfection plus 24-h exposure to 1.2 µM Nd(NO_3_)_3_ (full treatment group). Data are presented as the mean ± SE (*n* = 3 independent experiments). * *p* < 0.05, **** *p* < 0.0001 compared to the control group.

In addition, treatment with Nd(NO_3_)_3_ led to a significant increase in the content of the ferroptosis markers SOD and MDA (*t* (4) = 6.38, *p* = 0.0031, Figure 2F; *t* (4) = 22.06, *p* < 0.0001, Figure 2G) and a significant decrease in GSH levels (*t* (4) = 7.70, *p* = 0.0015, Figure 2H). qPCR and Western blot analyses revealed that Nd(NO_3_)_3_ significantly altered the gene and protein expression of circRNA_1156, ACSL4, LPCAT3, and PKCβII (*F* (3, 8) = 0.93, *p* = 0.0060; *F* (3, 8) = −0.85, *p* = 0.010; *F* (3, 8) = −0.43, *p* = 0.18; *F* (3, 8) = −1.04, *p* < 0.0001; *F* (3, 8) = −1.45, *p* < 0.0001; *F* (3, 8) = −1.14, *p* < 0.0001; *F* (3, 8) = −0.38, *p* = 0.02; *F* (3, 8) = 0.39, *p* = 0.02; *F* (3, 8) = −0.16, *p* = 0.42; *F* (3, 8) = −0.64, *p* = 0.0014; *F* (3, 8) = −3.23, *p* < 0.0001; *F* (3, 8) = −3.56, *p* < 0.0001; *F* (3, 8) = 0.50, *p* < 0.0001; *F* (3, 8) = −0.45, *p* < 0.0001; *F* (3, 8) = 0.02, *p* = 0.17; *F* (3, 8) = −0.38, *p* = 0.02; *F* (3, 8) = 0.39, *p* = 0.02; *F* (3, 8) = −0.16, *p* = 0.42; *F* (3, 8) = 0.0028, *p* = 0.99; *F* (3, 8) = −0.46, *p* < 0.0001; *F* (3, 8) = −0.58, *p* < 0.0001, as shown in Figure 3 and Figure 4). These results indicate that Nd(NO_3_)_3_ induces significant oxidative stress in hepatocytes, leading to ferroptosis.

However, overexpression of circRNA_1156 effectively reversed these effects. Compared with the group treated with Nd(NO_3_)_3_ alone, overexpression of circRNA_1156 significantly inhibited the gene and protein expression of ACSL4 and PKCβII (*p* < 0.01, Figure 3 and Figure 4), indicating that it alleviates ferroptosis by regulating the ACSL4/PKCβII pathway. This finding highlights the critical role of oxidative stress in mediating ferroptosis, as the overexpression of circRNA_1156 not only downregulated the ACSL4/PKCβII pathway but also reduced ROS levels and restored mitochondrial function, thereby mitigating oxidative stress-induced cell death.

However, overexpression of circRNA_1156 effectively reversed these effects. Compared with the group treated with Nd(NO_3_)_3_ alone, overexpression of circRNA_1156 significantly inhibited the gene and protein expression of ACSL4 and PKCβII (*p* < 0.01, Figure 3 and Figure 4), indicating that it alleviates ferroptosis by regulating the ACSL4/PKCβII pathway.

### 3.2. In Vivo Experimental Results

**Figure 5:**
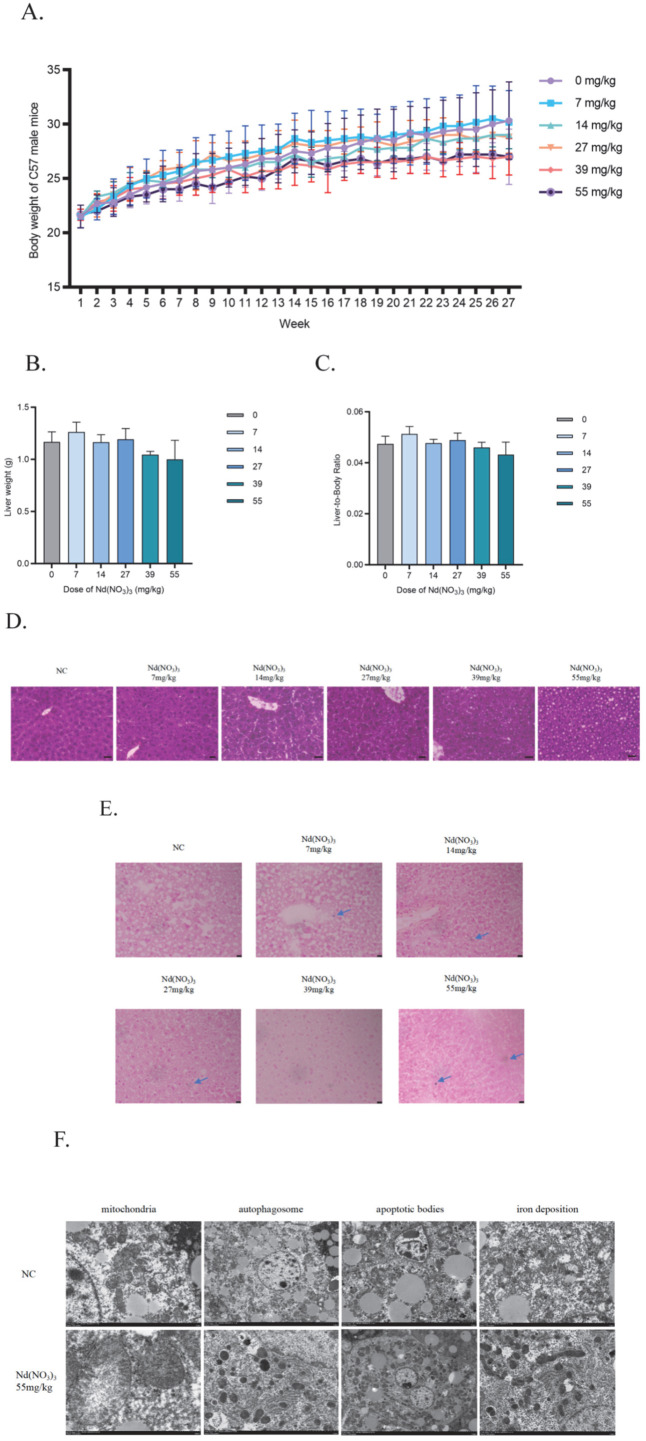
Effects of Nd(NO_3_)_3_ on body weight, liver weight, organ index, and pathological histology in C57BL/6J mice. (**A**) Changes in body weight over 180 days. (**B**) Absolute liver weight. (**C**) Liver-to-body weight ratio. (**D**) Hematoxylin and Eosin (H&E) staining of liver tissues. Scale bar = 100 µm. (**E**) Prussian blue staining for iron deposition in liver tissues. Magnified areas are indicated with arrows, and iron deposits are annotated. Scale bar = 50 µm. (**F**) Transmission electron microscopy of liver tissues. *Legend*: Liver tissues were stained with H&E to visualize cellular structures and with Prussian blue to detect iron deposits. Magnified areas highlight the presence of iron deposits, which appear as blue-black granules. Scale bars are included to provide a reference for the size of the structures shown. Data are presented as the mean ± SE (*n* = 6 independent experiments).

Long-term exposure to Nd(NO_3_)_3_ (7–55 mg/kg for 180 days) did not significantly decrease the body weight, absolute liver weight, or liver-to-body weight ratio in C57BL/6J mice (*p* > 0.05, Figure 5A–C).

Effects of Nd(NO_3_)_3_ on Liver Histology in C57BL/6J Mice: To enhance the clarity and interpretability of the liver histology data, we have added scale bars to each image. For Prussian blue staining, we have magnified the relevant areas and annotated the locations of iron deposits. These revisions improve the clarity and interpretability of the histological data. The results show that Nd(NO_3_)_3_ induces significant iron deposition in liver tissues, as evidenced by the presence of blue-black granules in Prussian blue-stained sections. Histopathological analysis of liver tissues did not show obvious inflammatory infiltration or structural damage (Figure 5D). Prussian blue staining further confirmed a significant increase in hepatic iron deposition in the Nd(NO_3_)_3_-exposed groups (Figure 5E). Transmission electron microscopy revealed that the mitochondria of hepatocytes in the Nd(NO_3_)_3_-treated mice exhibited typical ferroptosis features, including cristae disruption and membrane structural swelling (Figure 5F).

**Figure 6:**
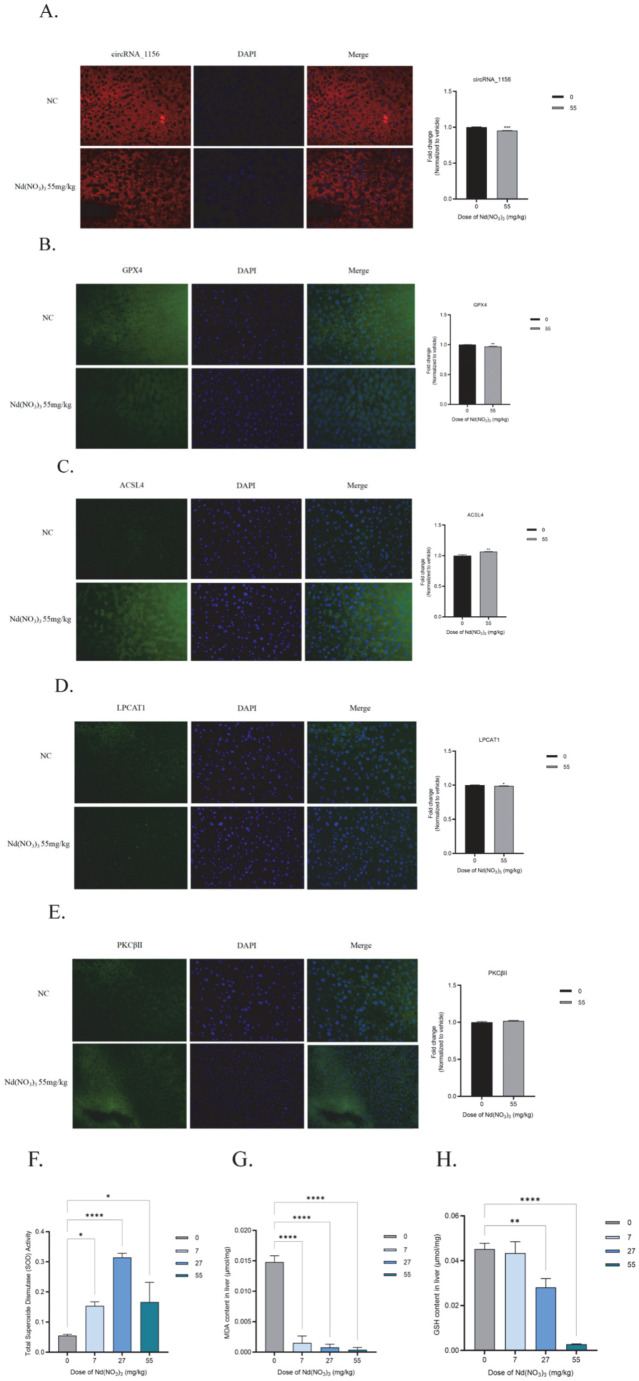
Effects of Nd(NO_3_)_3_ on fluorescence in situ hybridization, immunofluorescence, and ferroptosis biomarkers in C57BL/6J mice. (**A**) Fluorescence in situ hybridization (FISH) for circRNA_1156. (**B**) Immunofluorescence staining for GPX4. (**C**) Immunofluorescence staining for ACSL4. (**D**) Immunofluorescence staining for LPCAT1. (**E**) Immunofluorescence staining for PKCβII. (**F**) SOD activity levels. (**G**) MDA levels. (**H**) GSH levels. Data are presented as the mean ± SE (*n* = 3 independent experiments). * *p* < 0.05, ** *p* < 0.01, *** *p* < 0.001, **** *p* < 0.0001 compared to the control group.

Additionally, the protein expression of ACSL4 and PKCβII in liver tissues was continuously activated, while the fluorescence in situ hybridization (FISH) signal of circRNA_1156 was significantly downregulated (Figure 6A–E).accompanied by an initial increase followed by a decrease in superoxide dismutase (SOD) activity (*F* (3, 8) = −0.099, *p* = 0.02; *F* (3, 8) = −0.26, *p* <0.0001; *F* (3, 8) = −0.11, *p* = 0.01, Figure 6F), increased levels of malondialdehyde (MDA) (*F* (3, 8) = 0.013, *p* <0.0001; *F* (3, 8) = 0.014, *p* <0.0001; *F* (3, 8) = 0.014, *p* <0.0001, Figure 6G), and depletion of glutathione (GSH) (*F* (3, 8) = 0.0018, *p* = 0.89; *F* (3, 8) = 0.017, *p* = 0.0059; *F* (3, 8) = 0.042, *p* <0.0001, Figure 6H).

### 3.3. Comprehensive Results

Both cell and animal experiments consistently demonstrated that Nd(NO_3_)_3_ induces ferroptosis in hepatocytes by activating the ACSL4/PKCβII pathway. Overexpression of circRNA_1156 alleviates oxidative stress and lipid peroxidation by inhibiting this pathway, thereby exerting a hepatoprotective effect.

## 4. Discussion

Our study provides evidence that circRNA_1156 attenuates neodymium nitrate-induced hepatocyte ferroptosis by modulating the ACSL4/PKCβII signaling axis. While our findings suggest a regulatory role for circRNA_1156 in this pathway, we acknowledge the lack of direct evidence for transcriptional regulation of ACSL4 and PKCβII by circRNA_1156. Future studies are needed to elucidate the precise mechanisms by which circRNA_1156 affects the expression of these genes. While earlier studies have identified roles for circRNAs such as circRNA_104718 in modulating lipid metabolism through miRNA sponging (e.g., miR-29b-3p) [16,17], our findings uniquely demonstrate that circRNA_1156 can influence the ACSL4/PKCβII pathway, potentially through novel mechanisms distinct from canonical miRNA-dependent interactions. This represents a novel paradigm in circRNA biology, expanding their known functions beyond competitive endogenous RNA (ceRNA) mechanisms.

The association between ferroptosis and oxidative stress is well established in the literature. Ferroptosis is characterized by the accumulation of lipid ROS and depletion of GSH, leading to oxidative damage and cell death [2]. Our study further elucidates this relationship by demonstrating that Nd(NO_3_)_3_ induces ferroptosis through the ACSL4/PKCβII pathway, which is known to promote lipid peroxidation and oxidative stress [3,18,19]. The overexpression of circRNA_1156 effectively mitigates these effects by inhibiting the ACSL4/PKCβII pathway, thereby reducing ROS levels and restoring mitochondrial function. This highlights the critical role of oxidative stress in mediating ferroptosis and suggests that targeting the ACSL4/PKCβII pathway could be a promising therapeutic strategy for reducing oxidative stress-induced liver damage.

In addition to SOD and GSH, other specific markers of ferroptosis include malondialdehyde (MDA), which is a product of lipid peroxidation and commonly used to indicate ferroptosis. Ferritin degradation is also a characteristic of ferroptosis, as it reflects the release of iron from ferritin stores. Other markers such as 4-hydroxynonenal (4-HNE) and increased cellular iron levels can also be used to assess the occurrence of ferroptosis. Furthermore, in contrast to prior work focusing on GPX4 or System Xc^−^ as primary ferroptosis regulators in heavy metal toxicity, our data highlight the ACSL4/PKCβII pathway as a critical vulnerability in Nd(NO_3_)_3_-driven hepatic damage [16,17]. This divergence underscores the context-dependent nature of ferroptosis regulation, where rare earth elements may preferentially activate lipid peroxidation cascades over iron overload pathways.

Role of circRNA_1156 in Mitochondrial ROS Regulation: Our findings demonstrate that overexpression of circRNA_1156 leads to a significant decrease in mitochondrial ROS levels, suggesting that circRNA_1156 plays a protective role against oxidative stress. This effect may be attributed to the downregulation of the ACSL4/PKCβII pathway, which is known to promote lipid peroxidation and oxidative stress. By reducing mitochondrial ROS levels, circRNA_1156 overexpression may help maintain mitochondrial integrity and function, thereby alleviating ferroptosis.

Ferroptosis is implicated in liver disease pathogenesis. Here, we elucidate the role of circRNA_1156 in neodymium nitrate (Nd(NO_3_)_3_)-induced hepatocyte ferroptosis. Our findings identify circRNA_1156 as a novel inhibitor of Nd(NO_3_)_3_-triggered ferroptosis via ACSL4/PKCβII downregulation, offering therapeutic insights for rare earth element-induced hepatotoxicity.

Clinical Relevance of Nd(NO_3_)_3_ Toxicity: Nd(NO_3_)_3_, as a rare earth compound, has gained significant attention due to its potential biological toxicity. Long-term exposure to Nd(NO_3_)_3_ has been associated with liver damage and other health issues, including oxidative stress and lipid peroxidation. Our study provides novel insights into the mechanisms underlying

Nd(NO_3_)_3_-induced hepatotoxicity by demonstrating that Nd(NO_3_)_3_ triggers ferroptosis through the activation of the ACSL4/PKCβII pathway. This pathway is known to exacerbate oxidative stress and lipid peroxidation, leading to cell death.

Clinical Implications: The findings from our study highlight the critical role of oxidative stress in mediating Nd(NO_3_)_3_-induced liver damage. In clinical settings, understanding the mechanisms of rare earth element toxicity is essential for developing targeted therapies to mitigate liver injury.

Our results suggest that interventions aimed at inhibiting the ACSL4/PKCβII pathway could be a promising therapeutic strategy for reducing oxidative stress-induced liver damage. Additionally, the protective effect of circRNA_1156 identified in our study provides a new target for the intervention of rare earth element-induced hepatotoxicity.

These advances not only deepen mechanistic insights into ferroptosis but also bridge a critical gap in understanding how non-coding RNAs interface with environmental toxicant responses—a domain largely unexplored in prior literature [16,17]. Future studies should prioritize validating circRNA_1156’s binding partners (e.g., via RIP-seq) and assessing its translational potential in human models, steps essential to translating this preclinical novelty into therapeutic innovation.

**Figure 7:**
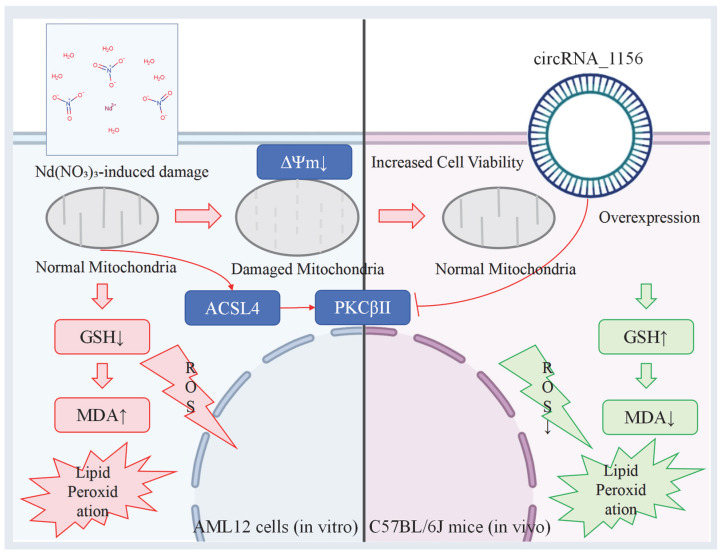
Molecular pathway diagram showing circRNA_1156 targeting and regulating the expression of ACSL4 and PKCβII to block ferroptosis. Legends: Color Definitions: Red arrows: Indicate the Nd(NO_3_)_3_-induced mitochondrial damage pathway (ROS↑ → MDA↑ → GSH↓ → ΔΨm↓); Green arrows: Represent the rescue effects of circRNA_1156 overexpression (ROS↓ → MDA↓ → GSH↑ → restored cell viability); Black arrows: Denote increases (↑) or decreases (↓) in specific indicators.

Our study confirmed that exposure to Nd(NO_3_)_3_ significantly activates the expression of ACSL4 and PKCβII and induces ferroptosis in hepatocytes through lipid peroxidation and oxidative stress, characterized by ROS accumulation, GSH depletion, and mitochondrial structural damage [20,21] (Figure 2, Figure 3, Figure 4, Figure 5 and Figure 6). This finding is consistent with previous studies, in which ACSL4 exacerbates lipid peroxidation by promoting the metabolism of polyunsaturated fatty acids, while PKCβII promotes ferroptosis by enhancing oxidative stress signaling pathways [3,18,19]. Notably, overexpression of circRNA_1156 significantly reversed the toxic effects of Nd(NO_3_)_3_, including inhibition of the ACSL4/PKCβII pathway, reduction of ROS levels, and improvement of mitochondrial function [22,23,24] (Figure 3 and Figure 4). This suggests that circRNA_1156 may alleviate ferroptosis by targeting and regulating the expression of ACSL4 and PKCβII, thereby blocking the core molecular events of ferroptosis (Figure 7).

Although this study has clarified the negative regulatory role of circRNA_1156 on ACSL4/PKCβII, the specific mechanisms underlying this regulation still need to be further explored. Previous studies have shown that circRNAs can regulate downstream signaling pathways by sponging miRNAs or directly binding to proteins. For example, circRNA_104718 activates ACSL4-mediated lipid metabolic disorder by sponging miR-29b-3p. In light of the results of this study, it is speculated that circRNA_1156 may inhibit the transcription or translation of ACSL4 and PKCβII through a similar mechanism. Future studies could use RNA immunoprecipitation (RIP) or dual-luciferase reporter assays to verify whether circRNA_1156 binds to specific miRNAs or proteins, thereby clarifying the specific pathways through which it regulates ACSL4/PKCβII [1,25,26].

The widespread use of rare earth elements and their associated biological toxicity are becoming increasingly contradictory, and ferroptosis, as a novel form of cell death, is gradually being recognized for its role in heavy metal-induced hepatotoxicity [27,28,29]. The protective effect of circRNA_1156 identified in this study provides a new target for the intervention of rare earth element-induced hepatotoxicity. Moreover, the ACSL4/PKCβII pathway, as a key regulatory axis of ferroptosis [30,31,32], may have potential for clinical translation [2,33,34]. Given the critical role of oxidative stress in mediating ferroptosis, our discovery of circRNA_1156’s hepatoprotective effects offers a previously unexplored strategy to counteract rare earth element toxicity. Existing interventions, such as ACSL4 inhibitors (e.g., ferrostatin-1), broadly target lipid metabolism but lack specificity. The PPARγ agonist rosiglitazone has been shown to exert anti-ferroptotic effects in non-alcoholic fatty liver disease models, suggesting that modulation of the PPARγ pathway may be a promising therapeutic strategy [35,36,37]. By contrast, circRNA_1156 overexpression achieves pathway-selective inhibition without disrupting global lipid homeostasis, addressing a key limitation of current approaches.

Limitations: One limitation of our study is the lack of direct evidence demonstrating that circRNA_1156 transcriptionally regulates ACSL4 and PKCβII. Although our data indicate a significant association between circRNA_1156 expression and the ACSL4/PKCβII pathway, further experiments are required to confirm the specific molecular interactions involved. Future studies should focus on identifying the direct targets of circRNA_1156 and elucidating the detailed mechanisms of its action in the context of ferroptosis. In the animal experiments, long-term exposure to Nd(NO_3_)_3_ did not result in significant changes in body weight or hepatic histopathology, which may be related to the exposure dose or insufficient observation time. Further extension of the experimental period or increased dosage is needed. The universality of circRNA_1156 in other types of hepatocytes or human models has not been assessed. Future studies could combine multi-omics analysis to screen for molecules interacting with circRNA_1156 and use gene knockout animal models to verify its function.

## 5. Conclusions

circRNA_1156 alleviates Nd(NO_3_)_3_-induced ferroptosis in hepatocytes by inhibiting the ACSL4/PKCβII pathway. This finding not only deepens the understanding of the toxicological mechanisms of rare earth elements but also provides a theoretical basis for the development of liver protection strategies. Our study highlights the critical role of oxidative stress in mediating ferroptosis and suggests that targeting the ACSL4/PKCβII pathway could be a promising therapeutic strategy for reducing oxidative stress-induced liver damage.

## Figures and Tables

**Table 1:** List of primers for quantitative real-time polymerase chain reaction (qRT-PCR).

Target Gene	GenBank^®^Accession Number	Primer Sequences (5′–3′) Forward (Fw) and Reverse (Rw)	Length (bp)	Annealing T (°C)	Fragment Size (bp)
*circRNA_1156*	NC_000077.7	F: GTAAGGTGTGGCTTCTGGCT	20	60	113
		R: CAGTTGGAAAGGTGCAAGGC	20		
*ACSL4*	NM_207625	F: CCTTTGGCTCATGTGCTGGAAC	22	60	5280
		R: GCCATAAGTGTGGGTTTCAGTAC	23		
*LPCAT3*	NM_145130	F: CCATCTCTTCCACACCTTCACG	22	60	1932
		R: GGATGAGGAACTGAAGCACGAC	22		
*PKCβII*	NM_002738	F: CCAAGATGACGATGTGGAGTGC	22	60	8010
		R: CTCCATCACAAAGTACAGGCGG	22		
*GAPDH*	NM_008084	F: CATCACTGCCACCCAGAAGACTG	23	60	1257
		R: ATGCCAGTGAGCTTCCCGTTCAG	23		
*β-actin*	NM_007393	F: CATTGCTGACAGGATGCAGAAGG	23	60	1935
		R: TGCTGGAAGGTGGACAGTGAGG	23		

**Table 2:** List of molecular weight size for all the Western blots.

Antibody Name	Molecular Weight Size
ACSL4	79 kDa
LPCAT1	59 kDa
PKCβII	71 kDa
GPX4	20/22 kDa
SLC7A11	37 kDa
β-actin	43 kDa
p53	53 kDa
COX2	20 kDa

## Data Availability

The datasets generated and analyzed during this study are available in the National Genomics Data Center (NGDC) repository under accession number OMIX010051. Additional data supporting the findings of this study are included in this article and its Supplementary Materials. Requests for further information should be directed to the corresponding author.

## Supplementary Materials

The following supporting information can be downloaded at https://www.mdpi.com/article/10.3390/antiox14060700/s1. Figure S1: Experimental Design Flowchart.
