# Supplementary figures and images for: CircRNA_1156 Attenuates Neodymium Nitrate-Induced Hepatocyte Ferroptosis by Inhibiting the ACSL4/PKCβII Signaling Pathway
Source: Antioxidants (Basel). 2025 Jun 9;14(6):700. doi: 10.3390/antiox14060700 (PMC12189531; doi:10.3390/antiox14060700)

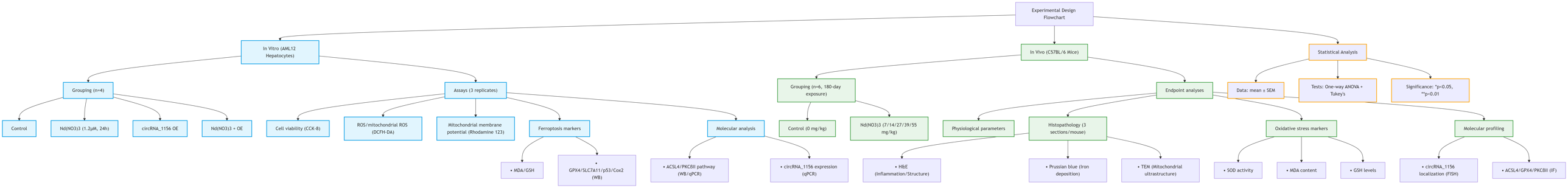

Supplement: Supplementary file 1 [file antioxidants-14-00700-s001.zip › antioxidants-3670620-supplementary.png]
